# Analytical results for integrate-and-fire neurons driven by dichotomous noise

**DOI:** 10.1186/1471-2202-14-S1-P243

**Published:** 2013-07-08

**Authors:** Felix Droste, Benjamin Lindner

**Affiliations:** 1 Bernstein Center for Computational Neuroscience, Berlin, 10115, Germany; 2 Institute for Physics, Humboldt-Universität zu Berlin, Berlin, 12489, Germany

## 

Models of the integrate-and-fire type have been widely used in the study of neural systems [1]. Usually, they consist of an evolution equation for the neuron's membrane voltage, complemented by a fire-and-reset rule that is applied once a voltage threshold is crossed. This minimalist description has allowed impressive analytical insights, for instance into neuronal information transmission properties [2], the effect of input correlations [3], or the dynamics of whole networks [4]. Further, it can be readily extended to include more complex behavior, such as spike-frequency-adaptation [5], which can then be studied in a well-understood setting.

The synaptic input to the neuron is usually modeled as a sequence of spikes with stochastic arrival times; mathematically speaking, it is a Poisson process where each event is a delta spike (shot noise). As such discrete input is notoriously difficult to treat analytically (but see [6]), many studies have employed the so called diffusion approximation, modeling the massive synaptic bombardment as Gaussian white noise.

**Figure 1:**
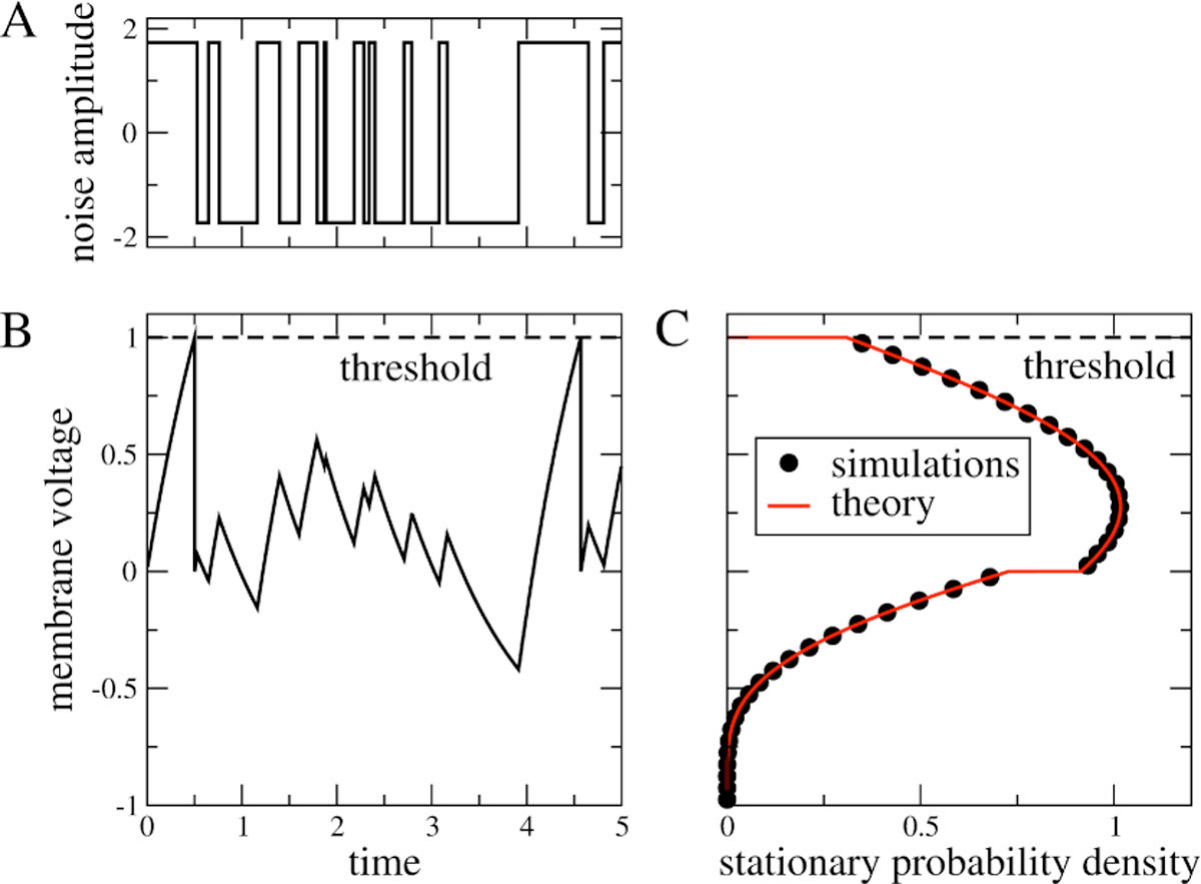
A) Sample time course of a two state noise. B) Corresponding voltage dynamics of a leaky integrate-and-fire neuron. C) Stationary distribution of this neurons membrane voltage. Units are arbitrary.

Here, we consider a general integrate-and-fire neuron that is driven by dichotomous noise, i.e. input that switches stochastically between two levels (cf. Figure 1A, see [7] for a similar setup). Input of this kind is of interest because it is temporally correlated, in contrast to the white noise often used. Also, when switching rates are asymmetric, it converges to excitatory shot noise in the limit of small correlation time. Further, it can model the switching between up and down-states of presynaptic network activity, or bursting activity of a presynaptic neuron.

We derive analytical expressions for firing-rate, CV and the steady-state voltage distribution of this system and verify them by numerical simulation. Furthermore, we study the transmission of a weak signal through such a neuron.
